# An Ultra-Wideband Frequency Selective Rasorber with Low Infrared Emissivity

**DOI:** 10.3390/ma17143414

**Published:** 2024-07-10

**Authors:** Hang Song, Yuning Zhang, Shengjun Zhang, Jingfeng Li, Xia Ai, Han Zhang, Jiaqi Liu

**Affiliations:** National Key Laboratory of Science & Technology on Test Physics and Numerical Mathematics, Beijing 100071, China; songhang0412@163.com (H.S.); jp2017303588@qmul.ac.uk (Y.Z.); zhangsj98@sina.com (S.Z.);

**Keywords:** frequency selective rasorber (FSR), sinusoidal microstrip lines, ultra-wideband, infrared shielding layer (IRSL), low infrared emissivity

## Abstract

The paper proposes an ultra-wideband frequency selective rasorber (FSR) with low infrared emissivity for the composite detection threat of both radars and infrared sensors. Firstly, the equivalent circuit (EC) method based on transmission line (TL) theory is utilized to analyze the absorption/transmission conditions. Then, based on the analysis above, sinusoidal microstrip lines with non-frequency-varying characteristics are adopted in the design, which significantly enhances the transmission bandwidth of FSR. The FSR demonstrates an absorption band ranging from 2.65 GHz to 8.80 GHz and a transmission band ranging from 9.15 GHz to 17.71 GHz. Furthermore, an infrared shielding layer (IRSL) exhibiting low emissivity in the infrared band and high transmittance in the microwave band is applied to the FSR. The simulation and experiment results verify that the IRSL-FSR demonstrates an ultra-wide transmission band ranging from 9.16 GHz to 17.94 GHz and an ultra-wide absorption band ranging from 2.66 GHz to 8.01 GHz. Additionally, it exhibits a low emissivity value (0.23) in 8–14 μm, providing a viable solution to the formidable challenge of radar-infrared bistealth for satellites and other communication-enabled flying platforms.

## 1. Introduction

Frequency selective rasorber (FSR) has garnered significant attention due to its potential in addressing the challenges associated with microwave transmission and stealth compatibility. The concept originated from a patent in 1995 [1], which asserts that the absorption and transmission of electromagnetic waves within a specific frequency band can be achieved through the placement of lossy material on a frequency selective surface (FSS) structure. The specific implementation concept was proposed by B.A. Munk in 2009 [2]. However, the actual structure was not provided for some reason. In 2012, F. Costa quantitatively derived the absorption and transmission conditions based on the equivalent circuit (EC) [3], which established a theoretical foundation for the structural design of FSR. Since then, numerous interesting findings on FSR have been published over the course of more than a decade of diligent research and development [4,5,6,7,8,9,10,11]. According to the relative position of the absorption band and the transmission band on the spectrum, three categories can be distinguished for FSR: low-transmission and high-absorption FSR (TA-FSR) [12,13,14,15,16], high-transmission and low-absorption FSR (AT-FSR) [17,18,19,20,21,22,23,24,25], and a transmission band between high and low-absorption bands (ATA-FSR) [26,27,28,29]. According to the unit structure dimension, FSR can be also classified into 3D-FSR [30,31] and 2D-FSR. While 3D-FSR offers greater design flexibility and structural strength, it poses challenges in processing, particularly in reducing the height of the longitudinal section, so there is a lack of mature application conditions for its implementation. Consequently, the current research primarily focuses on 2D-FSR.

Currently, the primary operating frequency range for a ground-based detection radar is 2–12 GHz, with a trend toward lower frequencies. Most micro-detectors on flight platforms such as satellites operate in the frequency band above 12 GHz, and there is a trend toward higher frequencies. Given this situation, AT-FSR, the metamaterial that incorporates high-frequency transmission and low-frequency absorption, has become an important focus for future stealth design. According to F. Costa’s equivalent circuit analysis theory [3], the AT-FSR requires a parallel resonant structure in order to achieve the high-frequency transmission window. However, expanding the bandwidth of the transmission window to meet practical application needs has become another challenge. Some designs promote using spiral microstrip lines instead of lumped inductors and capacitors, which can effectively reduce the insertion loss in the transmission band [18,19,20]. However, these results still do not meet the bandwidth requirements of micro-detectors. To address this issue, a design scheme based on densely arranged metal microstrip lines was proposed [29]. By perforating via holes in the dielectric plate of the RS and simultaneously loading metal microstrip lines densely on both sides of the dielectric plate connections, a significant broadening of the transmission bandwidth of AT-FSR is achieved. However, loading metal microstrip lines densely requires advanced processing technology and introduces significant processing errors; moreover, loading via holes can unpredictably affect the electromagnetic characteristics of the structure. Additionally, this design does not achieve broadband absorption while ensuring wideband transmission—further limiting its practical application value. Therefore, it is imperative to investigate the compatibility of AT-FSR in wideband transmission and absorption, in order to enhance its applicability in the stealth design of satellites and other aerospace platforms.

Furthermore, to achieve bistealth in microwave and infrared bands, two conventional approaches are employed. One approach involves the utilization of foam structures to achieve dual absorption of microwave and infrared electromagnetic waves, such as Ni−MXene/MF [32], Fe/3D carbon [33], and Fe/C/carbon foam [34]. The other entails coating the microwave absorber with low infrared emissivity materials like metal and nano-materials [35,36]. However, these methods fail to attain broadband absorption in the microwave band. In recent years, significant advancements in metamaterials have provided enhanced design flexibility for multi-spectrum composite stealth applications. Several multi-spectrum composite metamaterials with good absorption characteristics have been reported [37,38,39,40,41,42]. For example, Yang et al. developed a metamaterial utilizing quartz and ITO, which exhibits a 90% absorption rate within the frequency range of 5.1–19.2 GHz and an IR emissivity of 0.15 [41]. Zhang et al. proposed a transparent and flexible structure using ITO and polyvinyl chloride, achieving 90% absorption at 7.7–18 GHz with maintained stability at oblique incidence of 40 °C, while exhibiting an IR emissivity of only 0.23 [42]. Nevertheless, these aforementioned structures lack wave transmission functionality and cannot be applied to antenna system stealth designs.

In the past two years, there has been some research on designs featuring an infrared dual stealth communication radar [43,44,45,46]. For example, Zhang et al. introduced a metamaterial wave absorber based on graphene, which exhibits adjustable absorption performance and radar infrared composite stealth characteristics, showing promising application prospects [45]. However, this design lacks wave transmission functionality. Addressing this limitation, Fan et al.presented an actively tunable rasorber with broadband RCS reduction and low infrared emissivity. The transmission frequency can be continuously tuned from 1.8 to 4.5 GHz by applying a bias voltage of 0–15 V [46]. However, due to the absence of parallel resonance characteristics in the design structure, it cannot achieve a transmittance frequency higher than the absorption frequency. Additionally, while offering tunability through varac diodes, the structure also incurs significant wave penetration loss and narrow wave transmission bandwidth—rendering it unsuitable for high-efficiency communication scenarios in military applications.

To conclude, despite the distinctive and intriguing features demonstrated in the aforementioned studies, there remain certain limitations:

(1) In the current research of FSR, achieving wideband absorption and wideband transmission simultaneously is often challenging due to their inherent incompatibility.

(2) There is limited research on the integrated design of communication-radar infrared bistealth. From an engineering perspective, the existing design [46] has certain limitations: the center frequency of transmission does not align with the operating frequency of the micro detector, and the transmission bandwidth fails to meet the operating bandwidth requirements of the antenna.

In consideration of the constraints of the aforementioned study and the requirements for the practical implementation of micro detectors, the paper proposes an ultra-wideband FSR with low infrared emissivity. Firstly, by considering the absorption and transmission conditions based on the EC, we analyzed that impedance mismatch is essentially responsible for limiting the transmission bandwidth, and then we introduce a novel design approach utilizing non-frequency characteristic structures to optimize the imaginary part derivative of impedance and implement the design accordingly. By employing sinusoidal microstrip lines as parallel resonance structures, we achieve an ultra-wide transmission band along with an ultra-wide absorption band. Furthermore, the square film of indium tin oxide (ITO) with low infrared emissivity and high radar reflectivity was then applied onto the polyethylene glycol terephthalate (PET) substrate with a high filling rate, resulting in the formation of an infrared shielding layer (IRSL) characterized by low infrared emissivity and high radar transmission. By combining the IRSL with the aforementioned FSR, we successfully achieved a metamaterial possessing both wave transmission capabilities and stealth functions against radar-infrared detection. The proposed ultra-wideband (UWB) FSR exhibits an ultra-wide transmission band (9.01–16.05 GHz, $S_{21}\geq$ − 1 dB) and an ultra-wide absorption band (2.52–8.01 GHz, $S_{11}\leq$ − 10 dB). Additionally, it possesses a low emissivity value (0.26), enabling the transmission of radar waves and integrating radar-infrared bistealth functionalities.

## 2. Analysis, Design and Experimental Verification

**Figure 1:**
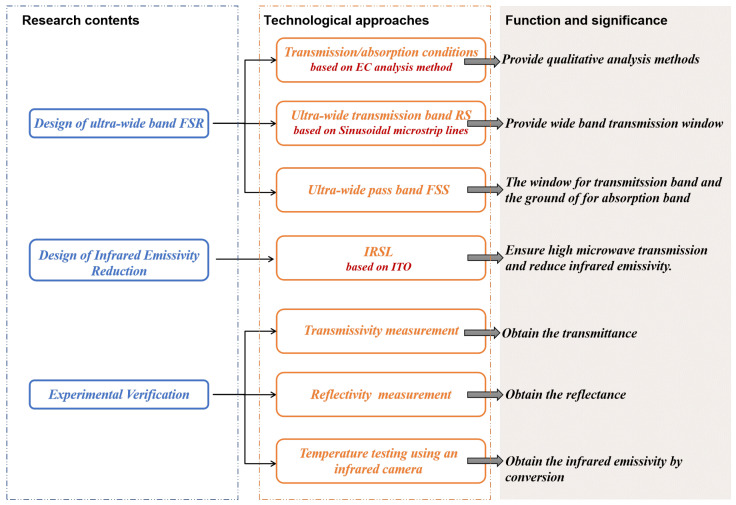
Framework of this paper.

Firstly, it is essential to clarify the technical approach adopted by this paper, as illustrated in Figure 1.

This paper establishes an analysis method suitable for FSR in Section 2.1 in order to gain a more intuitive understanding of its working principle. Subsequently, based on conclusions obtained from this analysis, RS and FSS are designed in Section 2.2. Subsequently, the two components are linked in a cascade fashion, and the design of an ultra-wideband FSR is achieved through adjustments to the interlayer spacing and resistance.

Building upon this foundation while considering wavelength differences between radar bands and infrared bands, an IRSL with a band-pass in the microwave band and band-stop in the infrared band was designed in Section 2.3, and then loaded onto the FSR. Through simulation and theoretical formulas, the absorption/transmission of IRSL-FSR within radar bands as well as emissivity within infrared bands are calculated.

Finally, in Section 2.4, the aforementioned FSR along with IRSL-FSR were processed and tested to verify the accuracy and effectiveness of the designs.

### 2.1. Transmission/Absorption Conditions Based on EC Analysis Method

The FSR is a composite structure comprising a resistive sheet (RS), a dielectric spacer (DS), and a band-pass FSS [2]. It ensures the proper transmission of signals within the working frequency band and absorbs, rather than reflects, out-of-band radar waves. This effectively reduces the omnidirectional RCS.

In addition, the FSR can be considered a combination of a circuit analog absorber (CAA) and a bandpass FSS [17]. According to the equivalent transmission line theory [3], the microwave system consisting of FSR and its surrounding free space can be represented as a two-port microwave network, with the dielectric layer and free space being equivalent to a two-wire transmission line. Port 1 and Port 2 serve as plane wave incident ports in free space. The lossy RS and lossless FSS are equivalent to circuit loads composed of resistors, capacitors, and inductors in parallel on the transmission line. The series resonance represented by *R*$L_{1}$$C_{1}$ in RS functions as low-frequency wave absorption, while $L_{2}$$C_{2}$ in RS and $L_{3}$$C_{3}$ in FSS represent parallel resonance, playing a role in high-frequency wave transmission.

**Figure 2:**
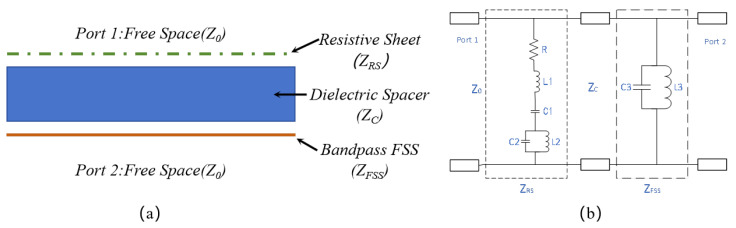
Sectional structure (**a**) and equivalent circuit of FSR (**b**).

Therefore, the structure and equivalent circuit can be illustrated as Figure 2, where $Z_{0}=120\;\pi \Omega$, represents the free-space impedance of Port 1 and Port 2. $Z_{RS}$, $Z_{C}$, and $Z_{FSS}$ denote the equivalent impedances of RS, DS, and FSS respectively. Actually, the intermediate DS typically employs a high transmission layer such as air or PMI foam, so it can be assumed that $Z_{c}=Z_{0}$.

According to transmission line theory, the ABCD matrix of the two-port network can be expressed as:

(1) $$\left[\begin{array}{cc} A & B\\ C & D \end{array}\right]=\left[\begin{array}{cc} 1 & 0\\ \frac{1}{Z_{RS}} & 1 \end{array}\right]\left[\begin{array}{cc} cos\delta & jZ_{0}sin\delta \\ j\frac{1}{Z_{0}}sin\delta & cos\delta \end{array}\right]\left[\begin{array}{cc} 1 & 0\\ \frac{1}{Z_{FSS}} & 1 \end{array}\right]$$

In Equation (1), $\delta =\frac{2\pi }{\lambda }tcos\theta$, while the incident wavelength is denoted as λ, the thickness of the DC layer is represented by *t*, and the refraction angle of the wave in DS is indicated as θ.

Therefore, the elements A, B, C, D in the matrix can be represented as follows:

(2) $$A=cos\delta +j\frac{Z_{0}}{Z_{FSS}}sin\delta$$

(3) $$B=jZ_{0}sin\delta$$

(4) $$C=\frac{Z_{RS}+Z_{FSS}}{Z_{RS}Z_{FSS}}cos\delta +j\frac{sin\delta cos\delta }{Z_{0}(1+1/Z_{RS}Z_{FSS})}$$

(5) $$D=cos\delta +j\frac{Z_{0}}{Z_{RS}}sin\delta$$

The transmission coefficient $S_{21}$ and reflection coefficient $S_{11}$ can be expressed as:

(6) $$S_{11}=\frac{AZ_{0}+B-CZ_{0}^{2}-DZ_{0}}{AZ_{0}+B+CZ_{0}^{2}+DZ_{0}}$$

(7) $$S_{21}=\frac{2Z_{0}}{AZ_{0}+B+CZ_{0}^{2}+DZ_{0}}$$

- ***Absorption conditions***

At the optimal absorption frequency $f_{a}$, the transmission coefficient $S_{21}$ and reflection coefficient $S_{11}$ of the two-port network are interrelated as follows: $\left|S_{11}\right|=0$, $\left|S_{21}\right|=0$.

In the above conditions, substitute Equations (2)–(5) into (6) and (7), respectively, and then we can obtain:

(8) $$Z_{RS}=sin^{2}\delta \left(Z_{0}+Z_{FSS}\right)-Z_{FSS}cos^{2}\delta -j\left(Z_{0}+2Z_{FSS}\right)sin\delta cos\delta$$

(9) $$\frac{Z_{0}Z_{FSS}^{2}}{tan\delta }+jZ_{0}Z_{FSS}\left(Z_{0}+Z_{FSS}\right)=0$$

According to Equation (9), $Z_{FSS}$ has two solutions:

(10) $$Z_{FSS}=\left\{\begin{array}{c} 0\\ -\frac{Z_{0}\left(tan\delta +j\right)}{tan\delta +1} \end{array}\right.$$

It is obvious that Re($Z_{FSS}$) can not be negative, so according to $Z_{FSS}$ = 0, FSS behaves similarly to a metallic ground plane at the frequency of absorption. Then, substitute Equation (10) into Equation (8):

(11) $$Z_{RS}=Z_{0}sin^{2}\delta -jZ_{0}sin\delta cos\delta$$

While $Z_{RS}=Z_{0}$, the incident wave enters DS through RS with no insertion loss, which is the condition of the premise of optimal absorption. Thus, we can easily find that:

(12) $$\delta =\frac{2\pi }{\lambda_{a}}tcos\theta =\frac{\pi }{2}$$

According to Equations (10) and (12), we can thus deduce the optimal absorption conditions.

1. FSS should be in a perfect stop band at $f_{a}$.
2. The equivalent thickness of the DC layer should be represented by $t_{0}=tcos\theta =\frac{\lambda_{a}}{4}$.

- ***Transmission conditions***

At the transmission frequency $f_{t}$, the transmission coefficient $S_{21}$ and reflection coefficient $S_{11}$ of the two-port network are interrelated as follows: $\left|S_{11}\right|=0$, $\left|S_{21}\right|=1$. Then, we can obtain the equations below according to Equations (6) and (7).

(13) $$\left(Z_{RS}+Z_{FSS}\right)-j\left(Z_{RS}-Z_{0}-Z_{FSS}\right)=0$$

(14) $$\left(2+\frac{Z_{0}Z_{RS}+Z_{0}Z_{FSS}}{Z_{RS}Z_{FSS}}\right)cos\theta +j\left(2+\frac{Z_{0}^{2}+Z_{RS}Z_{0}+Z_{FSS}Z_{0}}{Z_{RS}Z_{FSS}}\right)sin\theta =2$$

Equation (14) can evidently illustrate that $Z_{RS}Z_{FSS}\to \infty$. In addition, the expression of $Z_{RS}$ and $Z_{FSS}$ can be represented in terms of EC, as shown in Equations (15) and (16).

(15) $$Z_{RS}=R_{1}+\frac{1-\omega^{2}L_{1}C_{1}}{j\omega C_{1}}+\frac{j\omega L_{2}}{1-\omega^{2}L_{2}C_{2}}$$

(16) $$Z_{FSS}=\frac{j\omega L_{3}}{1-\omega^{2}L_{3}C_{3}}$$

(17) $$\frac{1}{w_{t}^{2}}=L_{2}C_{2}=L_{3}C_{3}$$

Then, substitute Equations (15) and (16) into Equation (13), and under the conditions of $Z_{RS}Z_{FSS}\to \infty$, it is clear to see that $Z_{RS}\to \infty$, $Z_{FSS}\to \infty$, which means both RS and FSS should be transparent at the transmission frequency $f_{t}$. And $Z_{RS}\to \infty$ indicates the absolute value of its imaginary part is also infinite, when $R_{1}$ is limited by absorption conditions. This means the absolute value of $L_{2}$ should be as large as possible, while the absolute value of $C_{2}$ should be extremely small according to Equation (17).

Further, it can be observed that the primary factor influencing the change in the transmission coefficient $S_{21}$ is that the imaginary part of $Z_{RS}$ mismatches rapidly in the spectral range around $f_{t}$. The discussion of high inductance and low capacitance above is only aimed at enhancing the transmission coefficient at the single frequency point $f_{t}$. In order to further broaden the wave transmission bandwidth, the key point is to maintain a high transmission coefficient over a wide spectral range around $f_{t}$. Thus, it is essential to identify a non-frequency-dependent resonant structure to mitigate the rapid mismatch of the imaginary part of the impedance.

Finally, the transmission conditions can be illustrated as follows:

1. Both RS and FSS should be transparent at the frequency transmission frequency $f_{t}$.
2. The resonant structure corresponding to $L_{2}C_{2}$ should maximize inductance and minimize capacitance to reduce insertion loss (IL).
3. The utilization of miniaturized resonant structures with non-frequency-varying characteristics may be a crucial method for broadening the wave transmission bandwidth, an aspect that has been overlooked in previous research.

### 2.2. Design of the FSR

According to the equivalent circuit analysis in Section 2.1, in order to create a transmission window with minimal insertion loss at a frequency higher than the absorption band, the equivalent circuit form of RS must include a parallel LC circuit. At the resonant frequency of the parallel LC circuit, the equivalent surface impedance of the impedance surface layer will approach infinity, ensuring low insertion loss for transmitted electromagnetic waves. For FSR’s bandpass FSS layer, it is necessary not only to be in a “bandpass” state within the wave-passing band of the RS layer but also to exhibit near-total reflection characteristics within its designed absorption band. Thus, the design of FSR should encompass both in-band transparency and out-of-band absorption characteristics. The two key challenges are as follows: first, designing wideband low insertion loss within the transmission band; secondly, achieving impedance matching across a wide absorption band. Therefore, FSR’s basic design concept is as follows: RS and FSS are designed for wideband transmission, respectively; subsequently cascading them and adjusting resistance values and interlayer spacing enables the realization of an FSR design featuring both broad wave absorption and transmission capabilities.

The conclusion in Section 2.1 indicates that the wideband transmission of RS is a prerequisite for achieving the overall FSR transmittance, thus necessitating the initial design of RS.

#### 2.2.1. Ultra-Wide Transmission Band RS Based on Sinusoidal Microstrip Lines

The key to achieving a wide transmission band in the region of $f_{t}$ = 11 GHz lies in the use of a non-frequency variable parallel-LC resonant structure with high inductance and low capacitance.

Sinusoidal microstrip lines are a type of structure with non-frequency-varying properties, commonly utilized in the design of UWB antennas [33]. This structure allows for stable electromagnetic performance over a wide band because of its self-similarity. However, there has been no research on the application of a non-frequency variable structure to a periodic structure; thus, it is necessary to investigate the impact of this structure in the wideband design of FSR.

**Figure 3:**
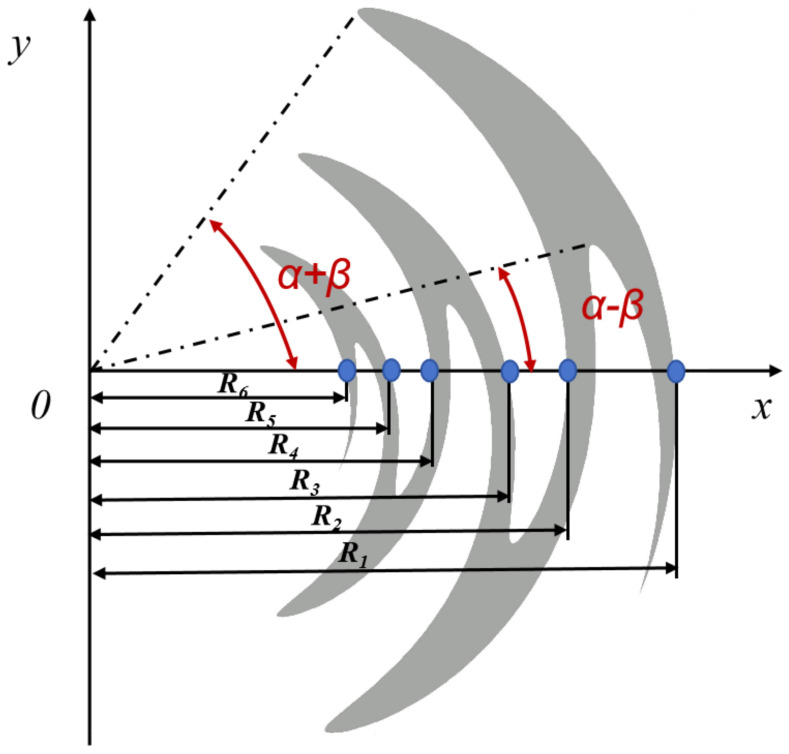
The structure of a sinusoidal microstrip line.

As depicted in Figure 3, the structure can be generated by a fundamental sinusoidal curve rotating β/2 in both clockwise and counterclockwise directions around the origin, with sinusoids comprising $r_{1}$, $r_{2}$, … $r_{n+1}$ and consisting of *n* curves whose size is determined by scaling factor τ and angle α. Among them, the n+1 curve can be obtained from the following equations:

(18) $$\varphi \left(r\right)={\left(-1\right)}^{n}\alpha sin\left[\frac{\pi ln(r/r_{n})}{ln\tau }\right];r_{n+1}\leq r\leq r_{n}$$

(19) $$r_{n+1}=\tau r_{n}$$

**Figure 4:**
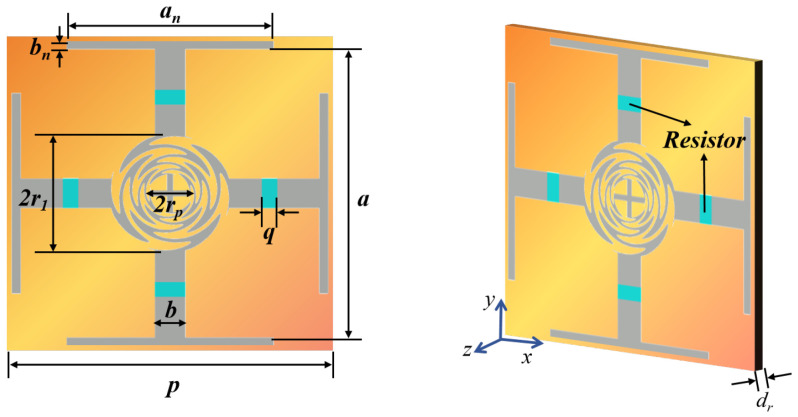
The structure of the RS unit cell.

The RS cell structure based on sinusoidal microstrip lines is illustrated in Figure 4. The metal layer (gray part) of the printed circuit board (PCB) was unilaterally loaded on a Rogers RT5880 substrate (orange part) with a thickness of $d_{r}$ = 0.127 mm, $\varepsilon_{r}$ = 2.2 and a dielectric dissipation factor of 0.0009. The Jerusalem cross of the four-arm sinusoidal microstrip line was loaded at the center of the metal layer unit cell. Each pair of sinusoidal microstrip lines is connected by a $2r_{n-1}$-long, 0.05 mm wide metal wire, and a 130 Ω patch resistor is loaded on each dipole of the Jerusalem Cross. The structural parameters are listed as follows: *p* = 8 mm, *a* = 7.6 mm, *b* = 0.8 mm, $a_{n}$ = 5 mm, $b_{n}$ = 0.1 mm, $r_{1}$ = 1.8 mm, α=π/4, β = α/4.5, τ = 0.8, *q* = 0.5 mm. The full-wave method utilized in this paper was proposed by CST 2022 with Floquet modes. The boundary conditions in the x–y direction are set as “unit cells”, while those in the z direction are “open (add space)”. The default options of Floquet modes are adopted for port and grid division. (This methodology is employed throughout all simulations in this paper and will not be reiterated in future instances).

**Figure 5:**
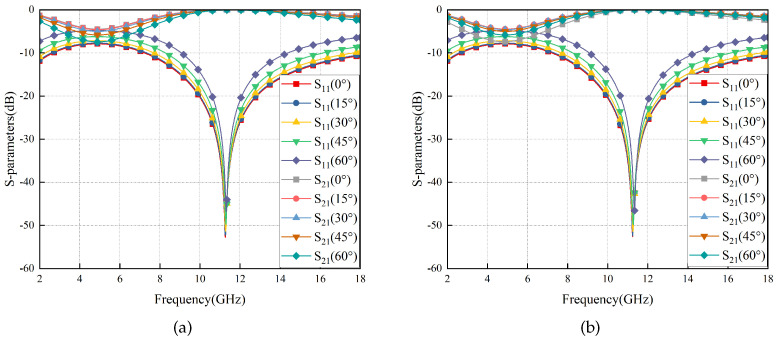
S-Parameters of the RS vary with ±60° TM (**a**) and TE (**b**) wave irradiation.

The simulation results are depicted in Figure 5, indicating that RS exhibits an exceptionally wide transmission band (8.72–16.80 GHz) and robust angular stability in ±60°. The curves of different colors/points represent the S-parameters at various incidence angles. For instance, the red square curve depicts the $S_{11}$ parameter at 0° incidence angle, while the gray square curve represents the $S_{21}$ parameter at 0° incidence angle. Similar information can be found in the figure’s legend for other curves. Additionally, the electromagnetic properties of RS remain almost identical when subjected to TE and TM wave irradiation, indicating its excellent polarization stability attributed to the strict central symmetry of the RS structure.

**Figure 6:**
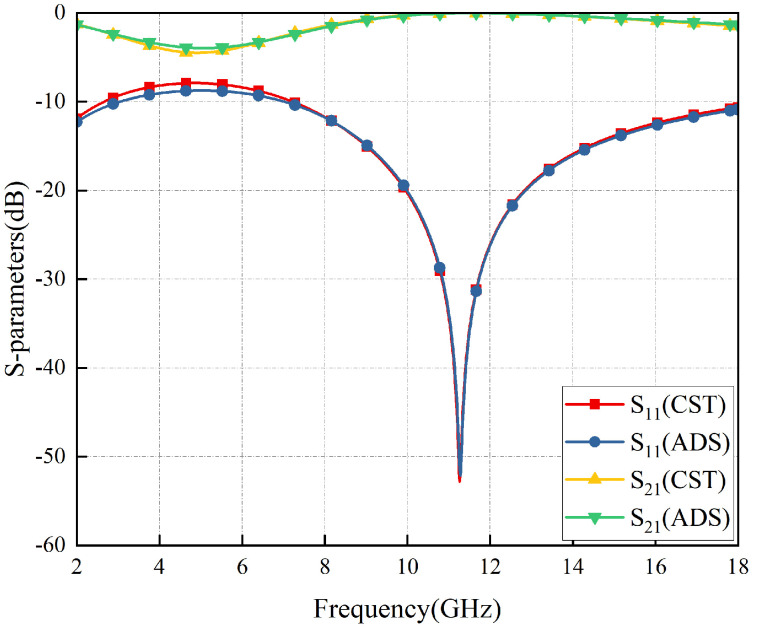
S-parameters of RS calculated by CST and ADS.

Furthermore, comparing the S-parameter results obtained by the full-wave simulation and the ADS equivalent circuit fitting, as shown in Figure 6, it is easy to see that the results are almost the same, which verifies the design concepts based on the EC analysis method proposed in this paper.

**Figure 7:**
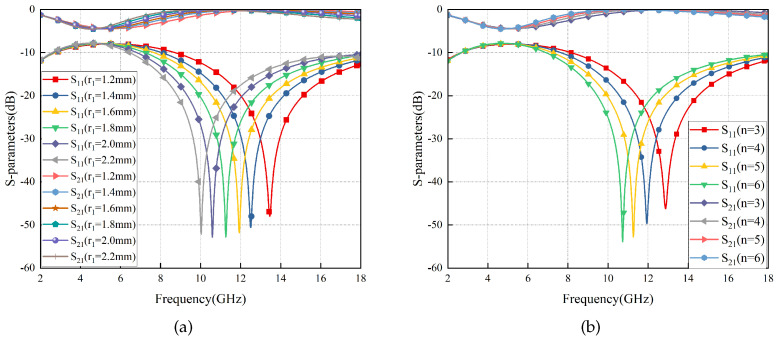
S-Parameters of the RS vary with $r_{1}$ (**a**) and $n_{1}$ (**b**).

It is noteworthy that the self-similar characteristics of the sinusoidal microstrip lines make the tuning of RS very simple. For instance, to adjust the wave transmittance performance, one only needs to modify the size of parallel LC in the EC by adjusting the corresponding structural parameter $r_{1}$ or *n*, as shown in Figure 7. As $r_{1}$ and *n* decrease, $f_{t}$ increases, and the curve shifts toward the high-frequency side. It is evident that smaller values of $r_{1}$ and *n* result in a narrower range from $r_{p}$ to $r_{1}$ and a narrower bandwidth of the non-frequency variable structure, which aligns with the inherent characteristics of a sinusoidal microstrip line structure, which also validates the accuracy of the second part of the conclusion on “Transmission Conditions” in Section 2.1. Therefore, considering the constraints of processing technology and the requirements of transmission bandwidth, *n* = 5 and $r_{1}$ = 1.8 mm were chosen.

In conclusion, this section presents the design of a resonator structure (RS) with an exceptionally wide transmission band based on a sinusoidal microstrip line. The RS demonstrates robust angular stability within ±60° and good polarization stability, with a transmission band ranging from 8.72 to 16.80 GHz. The full-wave simulation results of this design are consistent with those of the equivalent circuit model, which verifies the accuracy of the design. Thus, the RS provides a solid foundation for future research on wideband FSR.

#### 2.2.2. Flexible Ultra-Wide Passband FSS

The band-pass FSS is designed based on the relative position and bandwidth of the transmit-band of RS. According to the analysis in Section 2.1, the design of bandpass FSS should satisfy at least two requirements: firstly, it is crucial to ensure that the resonance frequency $f_{t_{2}}$ of FSS should align with $f_{t}$ in order to achieve optimal transmission; secondly, broadband total reflection should be achieved in the out-of-band frequency band to serve as a ground for low-band absorption design. This design approach is widely documented in the literature, such as in [17,18,19,22,23,24,25].

However, when evaluating FSR structures, $S_{21}\leq$ − 1 dB is typically considered the standard for high transmittance. Therefore, focusing solely on a single frequency point is insufficient. By analyzing the change trend of the $S_{21}$ curve in Figure 3, it is evident that the high-frequency side of the curve changes more gradually near the center frequency of transmission compared to the low-frequency measurement curve. Therefore, to ensure efficient utilization of the RS transmission band while maintaining a greater FSS passband than RS, it is necessary to ensure $f_{t_{2}}$ > $f_{t}$ when the FSS passband perfectly covers the RS transmission band.

Sandwich-type FSS with bilateral dielectric loading is an effective solution to achieving a wide passband. However, this method will lead to a significant increase in the thickness of FSS ($d_{f}$). In commonly used PCB flexible materials such as FR4, PTFE, Rogers RT5880, etc., when the thickness exceeds 0.4 mm, it will become a rigid medium. Therefore, FSS structure is faced with the contradiction between flexibility and broadband, which has also become the main factor restricting the conformity of FSR structures with a three-dimensional surface. Therefore, ensuring that the passband of FSS covers the transmission band of RS, while maximizing $f_{t_{2}}$ over $f_{t}$, is an effective approach to minimizing the thickness of FSS.

**Figure 8:**
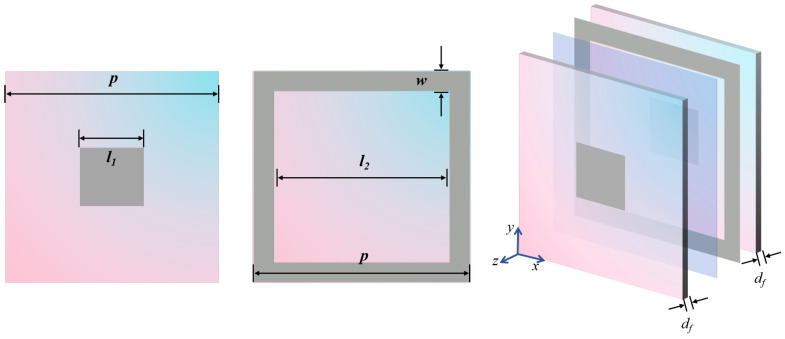
The structure of the FSS unit cell.

According to the aforementioned concepts, the FSS structure designed is illustrated in Figure 8. The dielectric substrate also utilizes Rogers RT5880 with $d_{f}$ = 0.127 mm, and the value of other structure parameters are shown as follows: *l* = 4 mm, $l_{f}$ = 3.1 mm, and $W_{f}$ = 1 mm. The two dielectric substrates are bonded with WL-PP280-045-50N semi-cured adhesive.

**Figure 9:**
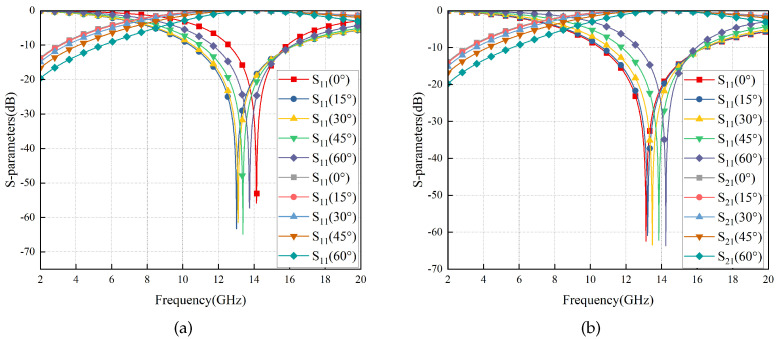
S-Parameters of the FSS vary with ±60° TM (**a**) and TE (**b**) wave irradiation.

The simulation results are depicted in Figure 9. The FSS shows good angular and polarization stability, and its passband (8.56–18.00 GHz) effectively covers that of RS (8.72–16.80 GHz) and ensures a wide wave transmittance bandwidth. Furthermore, it is evident that the FSS possesses a remarkably low thickness and exhibits flexibility, which is important to the conformal design.

#### 2.2.3. Ultra-Wide Transmission/Absorption Band FSR

The FSR structure is cascaded by RS and FSS. Under normal circumstances, a Floquet port corresponds to an RS unit of the same size as an FSS unit in order to facilitate simulation, leading to a slight difference between $f_{t_{2}}$ and $f_{t}$. This is not conducive to ensuring the wave transmission bandwidth and makes it difficult to reduce $d_{f}$. To address this issue, this paper proposes designing one RS unit for four FSS units. This approach can ensure sufficient simulation efficiency while effectively increasing the resonant frequency $f_{t_{2}}$ of FSS to reduce $d_{f}$.

**Figure 10:**
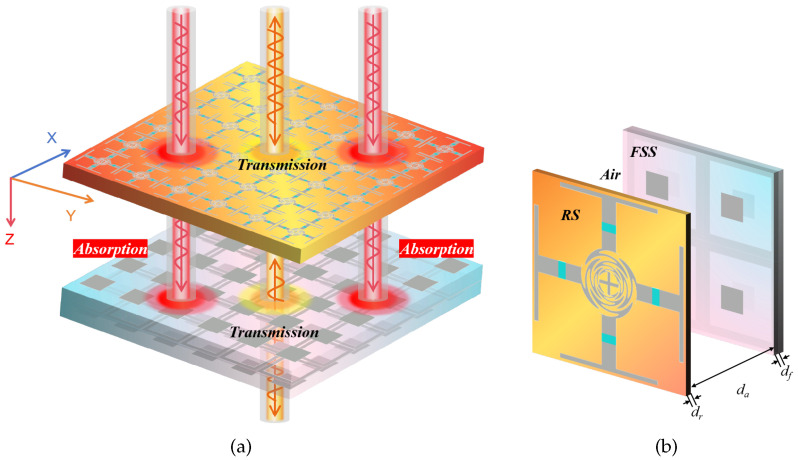
The structure and working principle of the whole FSR (**a**) and its unit cell (**b**).

Based on the aforementioned analysis, the RS and FSS designed above are cascaded and the DS between them is air, with a thickness of $d_{a}$ = 11 mm. Figure 10a depicts the structural schematic diagram of the FSR, while Figure 10b illustrates its unit cell.

**Figure 11:**
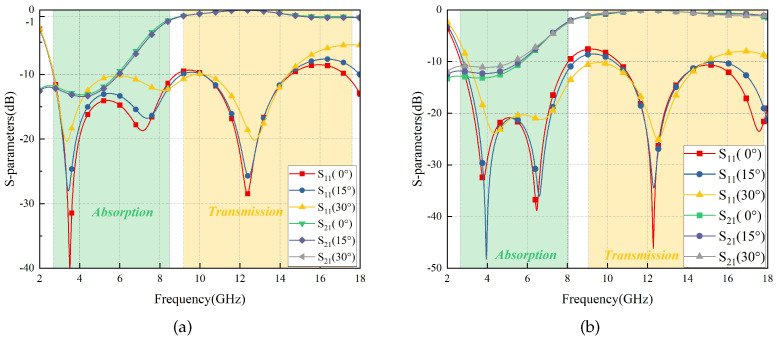
S-parameters of FSR vary with ±30° in TE polarization (**a**) and TM polarization (**b**).

The simulation results are depicted in Figure 11. According to relevant standards [1], $S_{21}\geq$ − 1 dB is considered to indicate high transmission performance, while $S_{11}\leq$ − 10 dB is considered to indicate high wave absorption performance. The FSR demonstrates a high absorption band of 107.4% relative bandwidth in the 2.65–8.80 GHz, and a high transmission band of 64.1% relative bandwidth in the 9.15–17.71 GHz. The FSR also shows good angular stability when the incident angle varies with ±30°.

Furthermore, due to the well-balanced symmetry, although the S parameters of the TE polarization are a little more stable than those of TM polarization, as is shown in Figure 11, the bandwidths are almost the same, so it can be considered that the FSR is polarization-insensitive.

**Figure 12:**
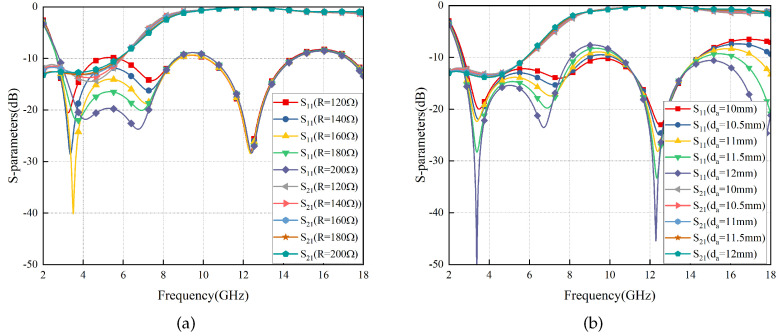
S-parameters of FSR vary with different *R* (**a**) and $d_{a}$ (**b**).

The influencing factors of the transmission effect have been discussed in Section 2.2.1, and now we will delve into the analysis of influencing factors affecting the absorption effect. According to the EC analysis method mentioned earlier, key determinants impacting FSR’s absorption include: DS thickness ($d_{a}$) and resistance value (*R*). Their respective effects on the absorption effect are shown in Figure 12.

With an increase in *R*, there is a noticeable increase in peak absorption rate within the absorption band; however, this also leads to a decrease in absorption bandwidth. The optimal compromise is achieved at *R* = 160 Ω.

Within a certain range, an increase in $d_{a}$ results in a decrease in minimum frequency within the absorption band; however, it also leads to a decrease in maximum frequency. Therefore, to ensure sufficient absorption bandwidth, $d_{a}$ = 11 mm is selected.

Additionally, it is noteworthy that based on the simulation results in Section 2.2.1 and Section 2.2.2, both RS and FSS exhibit miniaturization characteristics, with an angular stability of up to 60°. This is attributed to the effective miniaturization function of sinusoidal microstrip results, which effectively suppresses grating lobes. Furthermore, the 4:1 design of FSS and RS significantly reduces the unit size of FSS, thereby enhancing its angular stability.

**Figure 13:**
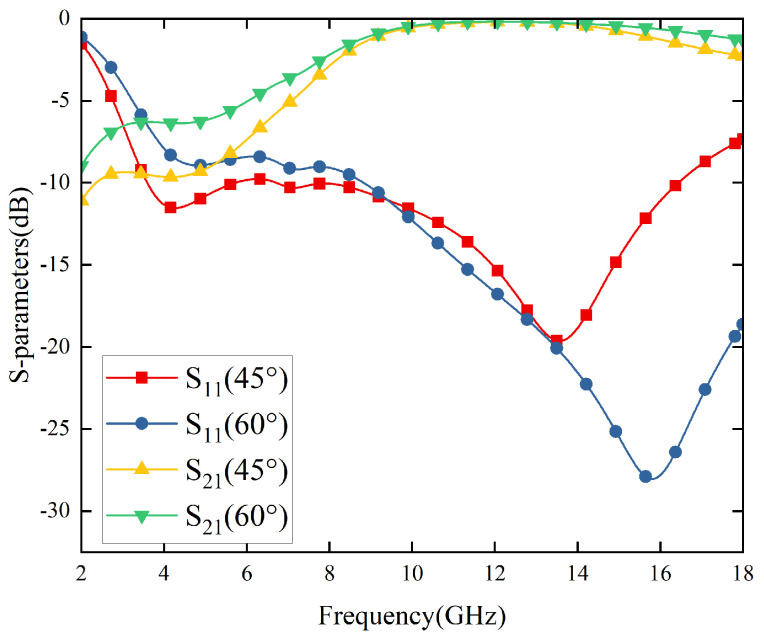
S-parameters of FSR vary with ±45° and ±60° in TM polarization.

However, as depicted in Figure 12, it can be observed that the angular stability of the FSR formed after cascading RS and FSS tends to deteriorate at 30°. Subsequently, using the TM wave as an example for simulating electromagnetic waves at oblique incidences of 45° and 60° (as shown in Figure 13), it was found that there is a significant reduction in transmittance bandwidth at 45° and poor low-frequency absorption effect at 60°—neither exhibiting high-performance ultra-wideband absorption/transmittance capabilities.

This phenomenon can be attributed to the fact that FSR functions as a sandwich-like multilayer structure equivalent to a circuit analog absorber (CAA) within the wave-absorbing segment. Under large angle incident wave irradiation, there are substantial changes in the equivalent thickness of the air layer between RS and FSS leading to noticeable deterioration in absorption effect (with more severe deterioration occurring with greater incidence angles). This elucidates why both RS and FSS demonstrate ±60° angular stability while the cascaded FSR does not possess such favorable characteristics.

### 2.3. Design of Infrared Emissivity Reduction

As long as the temperature of all forms of matter is above absolute zero, they emit infrared radiation in proportion to their temperature. Infrared emissivity is defined as the ratio of infrared energy emitted by an object at a certain temperature to that which would be emitted by a perfect black body at the same temperature [35]. The emissivity of a black body is 1, so the infrared emissivity of a substance can be understood as the ratio of its emitted infrared energy to that of a black body at the same temperature. Therefore, reducing the infrared emissivity can effectively reduce the infrared emission of the target, which is an important means of infrared stealth technology.

Due to the lower metal filling rate, the above-mentioned design of FSR obviously has a high infrared emissivity and therefore lacks infrared stealth capabilities. Below is the design for the low infrared emissivity of FSR.

The thermal radiation power of an object can be quantified as:

(20) $$M=e\sigma T^{4}$$

The infrared emissivity of the object is represented by *e*, while σ denotes the Stefan–Boltzman constant, and T stands for the temperature. Therefore, in the same environmental conditions, infrared emissivity can be utilized to characterize the thermal radiation power of an object and serves as an indicator for measuring its infrared stealth capability.

**Figure 14:**
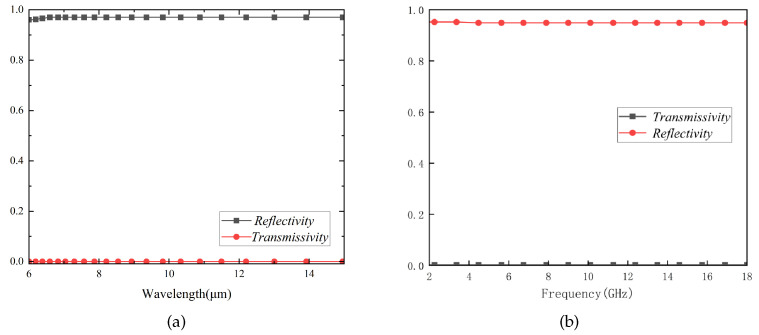
The transmissivity and reflectivity of ITO in the IR band (**a**) and microwave band (**b**).

As is shown in Figure 14, ITO is considered a Drude medium in the infrared band [5], with an extremely low emissivity (0.09). However, in the radar band, it also exhibits a very high reflectivity. Inspired by FSS design, to achieve high microwave transmittance, ITO’s nearly total reflection characteristic can be utilized as a substitute for metal as the unit cell pattern of FSS. The designed FSS has high transmittance in the microwave band and presents a first-order resonance band-stop characteristic in the infrared band, serving as an IRSL of the formerly designed FSR.

Furthermore, according to the following equation, where t represents the fill factor of ITO. To ensure a low $e_{IRSL}$, it is necessary to maximize the value of t as much as possible.

(21) $$e_{IRSL}=e_{ito}t+e_{sub}-e_{sub}t$$

**Figure 15:**
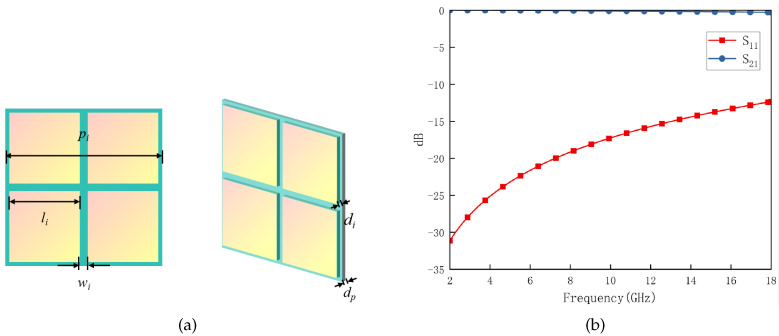
The structure of IRSL (**a**) and its S-parameters in the microwave band (**b**).

**Figure 16:**
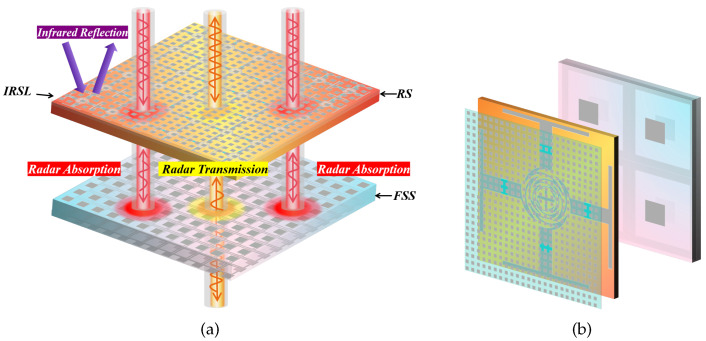
The structure and working principle of the whole IRSL-FSR (**a**) and its unit cell (**b**).

**Figure 17:**
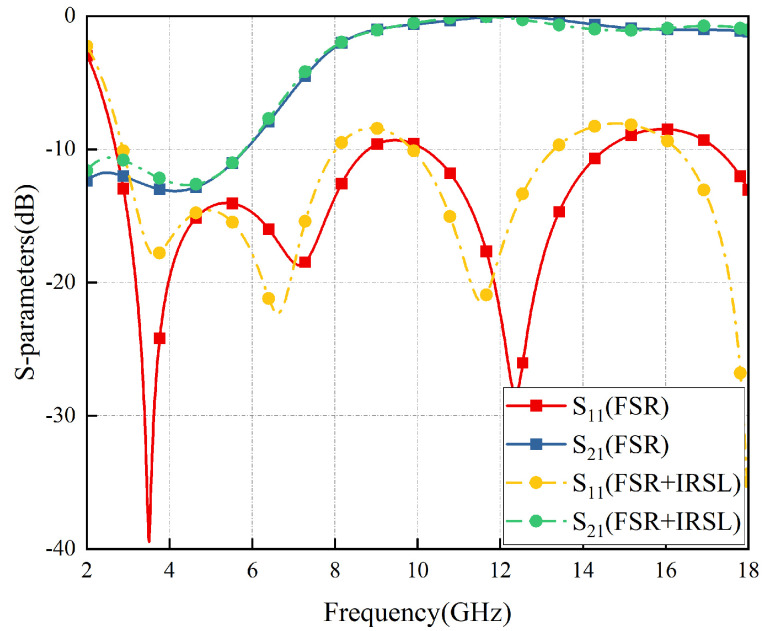
Comparison of S-parameters between FSR and IRSL-FSR.

Based on the above discussion, a $d_{i}$ = 300 nm thick ITO film with a sheet resistance of 6Ω/sq is printed on a $d_{p}$ = 0.125 mm polyethylene glycol terephthalate (PET) substrate with $e_{PET}$ = 0.9. The structure parameters in Figure 15a are presented as follows: $p_{i}$ = 0.8 mm, $l_{i}$ = 0.364 mm, $w_{i}$ = 0.036 mm, and the relative permittivity of PET is 3.0 (1 − 0.06j). As indicated in Figure 15b, this structure exhibits extremely high transmittance (nearly 99%) in the radar band. Furthermore, according to Equation (21), it can be inferred that $e_{IRSL}\approx$ 0.23. The IRSL is positioned 1 mm above the FSR to prevent electromagnetic interference, and both are surrounded by air as the intermediate medium. The unit cells of the them are cascaded at a ratio of 400:1 during simulation, as illustrated in Figure 16 for the structure and principle diagram and in Figure 17 for the simulation result. The results of simulation and calculation indicate that, after loading IRSL, the highest frequency of the absorption band of FSR decreases from 8.80 GHz to 8.01 GHz and the lowest frequency increases from 2.65 GHz to 2.66 GHz, with a slightly narrower bandwidth. Meanwhile, the transmission insertion loss increased from 0.02 to 0.045; however, the transmission band changed to 9.06–17.94 GHz. Therefore, it can be concluded that after loading IRSL, there is a slight modification between the absorption and transmission characteristics.

**Figure 18:**
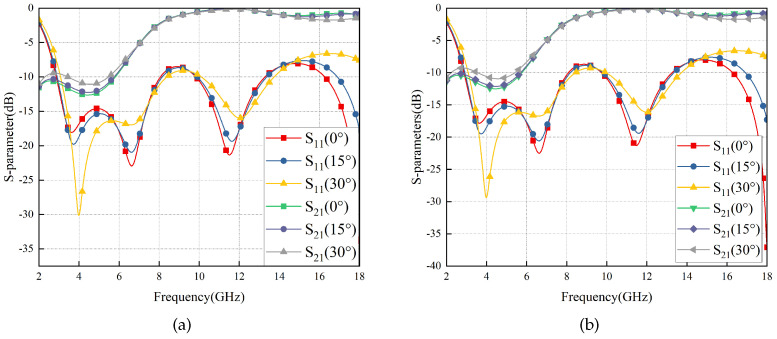
S-Parameters of the IRSL-FSR vary with ±30° TM (**a**) and TE (**b**) wave irradiation.

The S-parameters of IRSL-FSR under ±30° TM and TE waves were calculated, and the results are presented in Figure 18. It was observed that as the incident angle increased, there was a slight decrease in the bandwidth of the high-frequency transmittance band (greater than 15 GHz), while the low-frequency absorption effect (less than 3.8 GHz) deteriorated slightly. However, overall performance requirements were still met. Therefore, the introduction of IRSL has little impact on the polarization stability and angular stability of the structure.

In summary, an ultra-wideband IRSL-FSR with low infrared emissivity is designed in this section. The structure has an ultra-wideband absorption band of 2.66–8.01 GHz, an ultra-wideband transmission band of 9.06–117.94 GHz, and an infrared emissivity of 0.23 in the long-wave band. The design of broadband communication and radar infrared bistealth is realized.

### 2.4. Experimental Verification

The validity and accuracy of the IRSL-FSR and FSR designs mentioned above need to be verified through the following tests: (1) transmittance and reflectance tests in the microwave band (2–18 GHz); (2) infrared band (8–14 μm) temperature test, with conversion to their infrared emissivity.

The RS and FSS mentioned above were processed using printed circuit board technology. The metal material used was copper, and all dielectric substrates had a relative dielectric constant of 2.2, a loss tangent of 0.0009, and a thickness of 0.127 mm Rogers RT5880. The resistance model in the RS is a 0402 package with 160 Ω; the two layers of FSS were bonded together using a WL-PP280-045-50N semi-cured adhesive. To prevent the central collapse caused by the weight of RS, an 11mm polymethacrylimide foam (PMI) was added between RS and FSS to form a component.

**Figure 19:**
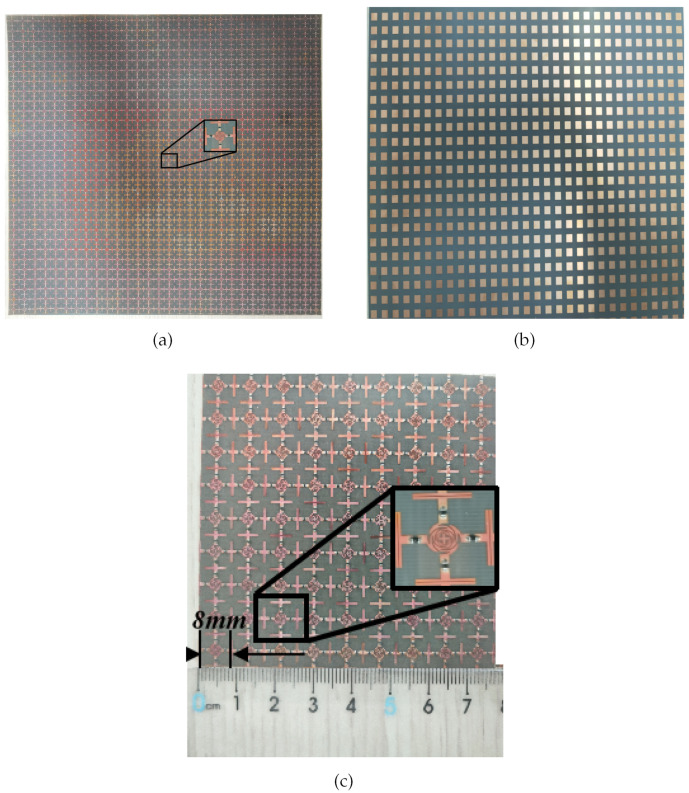
The structure of RS (**a**) and FSS (**b**), size of the unit cell (**c**).

In addition, considering the infinite size in FSR simulation and the finite size in actual machining, it is essential to minimize the truncation effect on the electromagnetic characteristics of the sample. Typically, engineering experience suggests that a noticeable truncation effect occurs when there are fewer than five unit cycles in both x and y directions but becomes negligible when there are more than 10 unit cycles. Therefore, a processed sample size of 300 mm × 300 mm standard parts was shown in Figure 19, with 37 unit cycles in both x and y directions.

**Figure 20:**
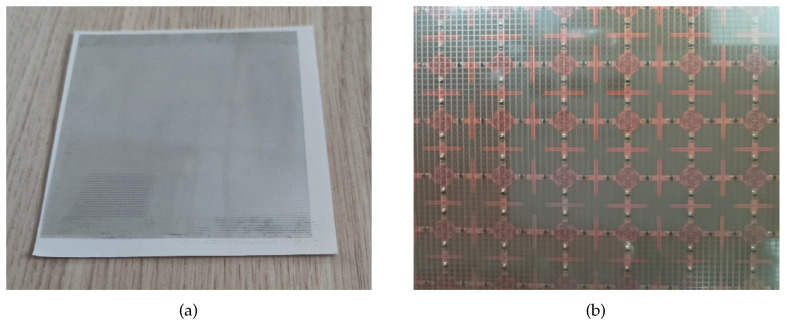
The structure IRSL (**a**) and IRSL-FSR (**b**).

Using magnetron sputtering technology, an ITO thin film with a thickness of 180 nm and sheet resistance of 6 Ω/sq was etched on a PET substrate with a relative dielectric constant of 3 (1 − j0.06), resulting in a 100 × 100 mm IRSL plate, as shown in Figure 20a. Subsequently, the IRSL was placed on a PET substrate with a thickness of 1 mm and cascaded with FSR to avoid electromagnetic interference between them. The IRSL was then placed on a 1 mm thick PET board and integrated with the FSR to mitigate potential electromagnetic interference. The IRSL-FSR sample is depicted in Figure 20b.

The samples above have been measured below.

**Figure 21:**
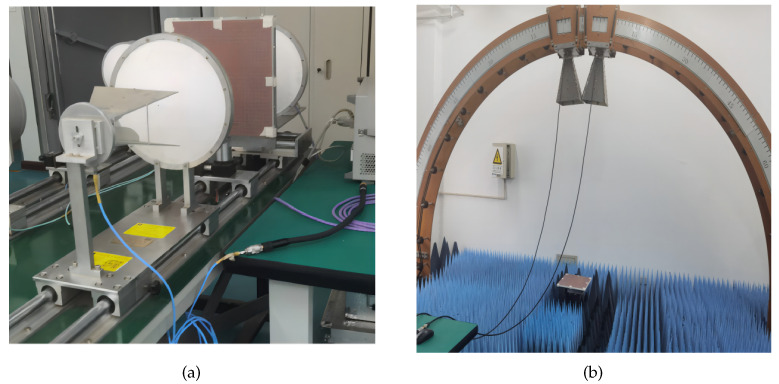
Transmissivity measurement (**a**), reflectivity measurement (**b**).

For the transmissivity measurement, the $S_{21}$ of FSR was tested using the free space test method. The experimental setup is illustrated in Figure 21a. In this configuration, pulse signals are transmitted via a transmitting horn antenna, received by a receiving horn antenna, and then analyzed using a vector network analyzer. During testing, the $S_{21}$ of the sample was measured both with and without placement on the fixture, and the sample’s $S_{21}$ was determined by taking their difference. The $S_{11}$ was determined using the bow frame test system. As depicted in Figure 21b, the sample and a metal plate of equal size were positioned at the center of the bow frame circle during testing, and the disparity between the two measurements was calculated using the time-domain gate function of the vector grid analyzer to derive the sample’s reflectivity.

**Figure 22:**
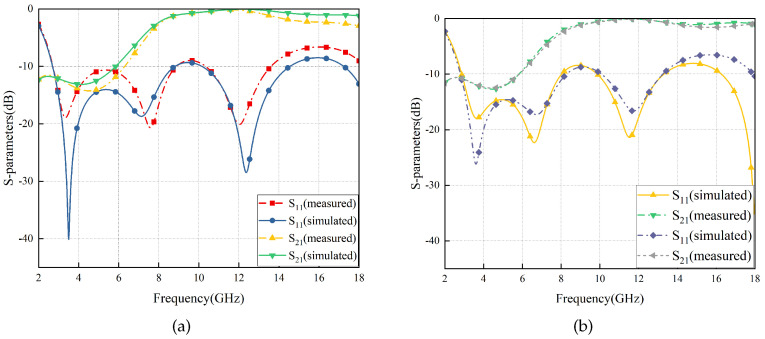
S-parameters comparison between measured and simulated FSR (**a**) and IRSL-FSR (**b**).

The comparison between the experimental results and the simulation results is shown in Figure 22. It can be seen that the two are generally consistent but with slight differences. The main reasons for the errors are the parasitic parameters caused by manual soldering of chip resistors and inherent errors in the testing system. In conclusion, the experimental results validate the accuracy of the simulation design.

**Figure 23:**
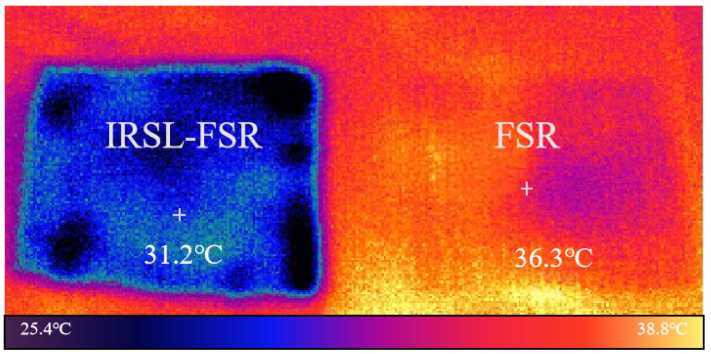
The IR imaging results at 40 °C.

Furthermore, the infrared emissivity of the verified samples are measured using an infrared camera. The FSR sample and the IRSL-FSR sample are both heated to the same temperature (40 °C). Subsequently, an infrared camera operating in the 8–14 μm range is used to capture images, and the resulting temperature data are shown in Figure 23. It can be observed that compared to FSR, IRSL-FSR significantly reduces the structure’s infrared emissivity. The infrared emissivity can be simulated by Equation (22), where $T_{r}$, $T_{a}$ and $T_{0}$, respectively, represent the temperature displayed in the IR image, the ambient temperature (25 °C), and the actual temperature (40 °C).

(22) $$e_{IRSL}=\frac{T_{r}^{4}-T_{a}^{4}}{T_{0}^{4}-T_{a}^{4}}$$

According to Figure 23 and Equation (22), the calculated emissivity of IRSL-FSR from testing is 0.26, which differs slightly from the calculated result of 0.23 in Section 2.3. This is due to uneven heating and the resistance welding of FSR. The calculated emissivity of FSR is 0.62; thus, the test results have validated that the design in this paper can effectively reduce the infrared emissivity of the structure.

Finally, a comparison between our designed structure and existing research findings was conducted, as is shown in Table 1. It was discovered that our design slightly improves the absorption bandwidth while greatly expanding the transmission bandwidth, all while maintaining the original low infrared emissivity. Additionally, even with the inclusion of IRSL, the structure designed in this paper still maintains a relatively minimal relative thickness.

## 3. Conclusions

In conclusion, this paper proposes an FSR structure with an ultra-wide absorption/transmission band and low infrared emissivity. Firstly, a method of expanding the FSR transmission bandwidth is proposed based on equivalent circuit analysis. This method utilizes RS based on a sinusoidal microstrip structure and cascade with FSS at 1:4 to form UWB FSR. Subsequently, an IRSL with low infrared emissivity and high radar transmittance is designed and combined with FSR. The experimental results demonstrate that after the loading of IRSL, the absorption band of FSR shifts from 2.65–8.80 GHz to 2.66–8.01 GHz, and the transmission band shifts from 9.15–17.71 GHz to 9.16–17.94 GHz, both exhibiting ultra-wideband characteristics. It also shows a low emissivity value (0.26) in 8–14 μm. Furthermore, the RS, FSS, and IRSL are all flexible, allowing for attachment to curved surfaces to achieve conformal design, and the thickness of IRSL-FSR is only 0.10 $\lambda_{L}$ ($\lambda_{L}$ is the lowest frequency on the spectrum and corresponds to the wavelength). In general, this research provides a viable solution to the challenge of radar-infrared bistealth for satellites or other communication-enabled platforms.

## 4. Primary Innovation

(1) In terms of research significance, the IRSL-FSR proposed in this paper represents a groundbreaking achievement in the integrated design of high-performance communication, radar stealth, and infrared stealth. This advancement effectively enhances the survivability of current flight communication platforms such as satellites/aircraft under the threat of ground-based radar + space-based infrared sensor multi-spectrum detection. As a result, it has significant potential applications in the military field.

(2) In terms of research methods, this paper proposes an FSR design method based on the non-frequency variable parallel resonant structure. Compared with the current dense metal wire layout design scheme, this method can significantly reduce processing requirements and achieve broadband absorption while ensuring a wide transmission band. This reflects good engineering value and can provide references for the wideband transmission design of FSR.

## Figures and Tables

**Table 1:** Comparison of this research to previously reported works.

References	−10 dB Absorption Band/Relative Bandwidth	−1 dB Transmission Band/Relative Bandwidth	Thickness/$\lambda_{L}$	Low IR Emissivity
Ref. [17]	2.80–8.30 GHz/99.0%	9.30–10.1 GHz/8.2%	0.112	No
Ref. [18]	2.40–7.10 GHz/98.9%	8.30–11.07 GHz/34.0%	0.166	No
Ref. [19]	3.00–8.90 GHz/100.1%	9.00–12.63 GHz/33.3%	0.105	No
Ref. [20]	2.10–6.20 GHz/98.7%	7.90–12.80 GHz/47.3%	0.110	No
Ref. [21]	3.20–8.70 GHz/92.4%	9.60–10.10 GHz/5.07%	0.121	No
Ref. [22]	2.35–6.78 GHz/97.0%	9.30–13.45 GHz/36.4%	0.118	No
**This paper (FSR)**	**2.65–8.80 GHz/107.4%**	**9.15–17.71 GHz/63.7%**	**0.099**	**No**
**This paper (IRSL-FSR)**	**2.66–8.01 GHz/100.3%**	**9.16–17.94 GHz/64.8%**	**0.100**	**Yes**

## Data Availability

The original contributions presented in the study are included in the article, further inquiries can be directed to the corresponding author.
